# White matter deficits correlate with visual motion perception impairments in dyslexic carriers of the DCDC2 genetic risk variant

**DOI:** 10.1007/s00221-021-06137-1

**Published:** 2021-07-06

**Authors:** Daniela Perani, Paola Scifo, Guido M. Cicchini, Pasquale Della Rosa, Chiara Banfi, Sara Mascheretti, Andrea Falini, Cecilia Marino, Maria Concetta Morrone

**Affiliations:** 1 Vita-Salute San Raffaele University, Milan, Italy; 2 C.E.R.M.A.C. (Centro di Risonanza Magnetica ad Alto Campo), Milan, Italy; 3 Nuclear Medicine Department, IRCCS San Raffaele Scientific Institute, Milan, Italy; 4 Institute of Neuroscience, National Research Council (CNR), Pisa, Italy; 5 Unit of Neuroradiology, IRCCS Ospedale San Raffaele, Milan, Italy; 6 Institute of Psychology, University of Graz, Graz, Austria; 7 Child Psychopathology Unit, Scientific Institute Eugenio Medea, Bosisio Parini, Italy; 8 Department of Psychiatry, Unviersity of Toronto, Toronto, Canada; 9 Division of Child and Youth Psychiatry, Centre for Addiction and Mental Health (CAMH), Toronto, Canada; 10 Department of Translational Research on New Technologies in Medicine and Surgery, University of Pisa, Pisa, Italy; 11 Scientific Institute Stella Maris (IRCSS), Pisa, Italy

**Keywords:** Dyslexia, DCDC2 gene, Psychophysics, Motion perception, White matter

## Abstract

Motion perception deficits in dyslexia show a large intersubjective variability, partly reflecting genetic factors influencing brain architecture development. In previous work, we have demonstrated that dyslexic carriers of a mutation of the *DCDC2* gene have a very strong impairment in motion perception. In the present study, we investigated structural white matter alterations associated with the poor motion perception in a cohort of twenty dyslexics with a subgroup carrying the DCDC2 gene deletion (DCDC2d+) and a subgroup without the risk variant (DCDC2d–). We observed significant deficits in motion contrast sensitivity and in motion direction discrimination accuracy at high contrast, stronger in the DCDC2d+ group. Both motion perception impairments correlated significantly with the fractional anisotropy in posterior ventral and dorsal tracts, including early visual pathways both along the optic radiation and in proximity of occipital cortex, MT and VWFA. However, the DCDC2d+ group showed stronger correlations between FA and motion perception impairments than the DCDC2d– group in early visual white matter bundles, including the optic radiations, and in ventral pathways located in the left inferior temporal cortex. Our results suggest that the DCDC2d+ group experiences higher vulnerability in visual motion processing even at early stages of visual analysis, which might represent a specific feature associated with the genotype and provide further neurobiological support to the visual-motion deficit account of dyslexia in a specific subpopulation.

## Introduction

Dyslexia is a heritable neurodevelopmental condition, affecting between 3 and 15% of the population, characterized by a specific and persistent failure to acquire reading skills, despite normal intelligence and adequate educational opportunities (Peterson and Pennington 2015). Current theories propose that dyslexia may originate from deficits in phonological processing (Landerl et al. 2013), auditory processing (Tallal 1980; Wright and Conlon 2009), visual attention (Facoetti et al. 2006, 2010) or visual perception (Stein and Walsh 1997).

Early evidence on post-mortem studies has suggested that the dyslexic brain includes several micro-alterations of the cortical structure supporting the hypothesis of a disturbance of neuronal migration (Drake 1968; Galaburda and Kemper 1979; Humphreys et al. 1990). In particular, some of these studies have pointed to a selective deficit of the magnocellular-dorsal system (Galaburda and Kemper 1979; Galaburda et al. 1985; Livingstone et al. 1991). Consistently, many studies have demonstrated deficits in visual processing of moving objects in subjects with reading impairments. One of the most robust findings is a sensitivity deficit for stimuli that require integrity of magnocellular pathway such as flickering gratings at a high temporal frequency (Martin and Lovegrove 1988; Cornelissen et al. 1995; Lovegrove 1996; Stein and Walsh 1997; Demb et al. 1998). However, the complete deficits of perception of visual motion is still elusive (Skottun 2005). For example, little consensus exists on whether dyslexics have impaired sensitivity to detect motion direction at low spatial frequencies, the optimal range for magnocellular processing, (up to 2 c/deg) (Martin and Lovegrove 1988; Cornelissen et al. 1995) or whether the deficits are limited to motion direction of high spatial frequencies (Cornelissen 1993; Slaghuis and Ryan 1999; Stuart et al. 2001). Deficits in direction discrimination of coherent random dot kinematograms (RDK) have also been reported (Cornelissen et al. 1995; Raymond and Sorensen 1998; Witton et al. 1998; Talcott et al. 2000; Hill and Raymond 2002; Pellicano and Gibson 2008; Benassi et al. 2010; Cicchini et al. 2015; Scerri et al. 2017), pointing to an impairment of associative motion cortices like V5/MT + , that are able to detect and classify the motion direction of these type of stimuli. However, the observed deficits of coherence sensitivity of these stimuli in dyslexia are very small. This may reflect the fact that the low spatial frequency information, that is prevailing in the RDK, is used to perform the task (Morrone et al. 2008; Cicchini et al. 2015). In addition, measurements of motion direction coherence sensitivity, gauged with RDK stimuli, require rather long exposure times (typically above 500 ms), compared to contrast direction sensitivity (about 100 ms), implicating also possible deficits in eye movements.

Currently, it is not clear whether the impairments are due to alterations in the processing of visual information in primary visual cortex, which typically impairs the detection of low contrast visual stimuli, or also to other intracortical mechanisms such as surround inhibition, that shape neural responses at higher level of motion pathways (like in MT). The effect of surround inhibition is particularly evident in the performance of motion discrimination at high contrasts (Tadin et al. 2003; Tadin 2015) for simple drifting sinusoidal gratings.

Importantly, many studies have reported weak average effects across large populations of dyslexics and a considerable variability between individuals (Hogben 1996; Spinelli et al. 1997; Amitay et al. 2002; Ramus et al. 2003; Roach et al. 2004; Wright and Conlon 2009; Talcott et al. 2013), making it unlikely that visual perceptual deficits of dyslexics are a shared trait of the whole population, calling for a segmentation of dyslexia in sub-types (Ramus et al. 2003; Skiba et al. 2011; Talcott et al. 2013).

In line with this view, recent work from our group has demonstrated that partitioning dyslexics into two subgroups—carriers and non-carriers of a deletion in DCDC2 gene—accounts for some of the variability of motion perception in dyslexics: carriers of the mutation displayed marked deficits at high spatial frequencies (4 c/deg), while the other poor readers displayed much milder deficits (Cicchini et al. 2015). Cicchini et al. investigated all possible visible range of drifting sinusoidal grating of different spatial and temporal frequencies and found that only the high spatial (above 1 c/deg) and temporal frequency (8 Hz) reveal profound deficits, even motion blindness in some subjects. In the same subjects, the RDK discrimination, which impinge on multiple spatio-temporal frequency channels, proved much less selective in demonstrating a difference between the same experimental subjects. Indeed, a recent study which attempted to measure the role of DCDC2 deletion on motion perception using RDK reported only a small effect between dyslexics and typicals and failed to detect a significant effect of genetic background (Scerri et al. 2017). Besides the employment of RDK, it is possible that other factors in the study of Scerri et al. contributed not to detect the motion deficit. In their work, they used white dots on black background presented on LCD screens which usually produces long screen persistence (even beyond 30 ms), presentation times of 2.5 s that allows many eye movement and image displacement every 50 ms that allows to use other cues, in addition to motion direction, to perform the task. All these parameters are clearly not optimal to investigate motion perception. Indeed, our previous results showed that the motion deficit can be observed only at high spatial frequency, at short exposure where eye movement cannot play a role, and at low contrast: these are the stimulus characteristics where motion detectors are particularly fragile in detecting direction. Corroborating our observation in humans, murine models of the same genetic alterations show impairments in motion perception (Rendall et al. 2017), together with other sensory deficits (Gabel et al. 2012; Truong et al. 2015; Centanni et al. 2016), suggesting that dyslexics carriers of DCDC2 mutation may constitute a specific subtype of dyslexia where the sensory deficits are a distinctive feature. Given the importance of the issue, we present here evidence that the DCDC2 deletion have deficit in white matter that correlates with the motion deficit, strengthening the suggestion that dyslexia phenotypes with DCDC2 alteration may have different behavioral and brain structure and should be subclustered in the large population of poor readers.

To date, several risk loci and four candidate genes have been identified for dyslexia [DXY1C1, KIAA0319, DCDC2 and ROBO1—(Taipale et al. 2003; Cope et al. 2005; Hannula-jouppi et al. 2005; Meng et al. 2005)], all of which are strongly implicated in neural migration during development (Galaburda et al. 2006), suggesting that dyslexics brain may present altered connections between brain areas. Several studies have linked reading disability with white matter anomalies in temporo-parietal regions part of the reading network such as the corona radiata (CR), arcuate fasciculus (AF) (Vandermosten et al. 2012) and the corpus callosum (von Plessen et al. 2002; Niogi and McCandliss 2006; Odegard et al. 2009). Importantly, in subjects with familial risk for dyslexia, some of these alterations are presented even before reading acquisition begins (Vandermosten et al. 2015; Wang et al. 2016; Vanderauwera et al. 2017).

Marino et al. (2014) investigated, by means of Diffusion Tensor Imaging (DTI), white matter alterations in poor readers carrying the DCDC2 intron 2 deletion. The researchers found clusters of alterations in the superior longitudinal, arcuate, inferior longitudinal fasciculi and corpus callosum, which are dorsal and ventral white matter tracts known to be part of the reading network (Vandermosten et al. 2012; Wandell and Yeatman 2013). Notably, Marino et al. (2014) reported also structural alterations in the splenium and the optic radiations. This finding is consistent with the hypothesis that the DCDC2 deletion might disrupt white matter organization in specific tracts that transfer the visual information necessary to mediate reading. The results of differential alteration of FA in the carriers of the DCDC2 deletion (Marino et al. 2014) together with the results from Cicchini et al. (2015) of a different sensitivity for motion in the two populations suggest that motion perception deficits may reflect white matter alteration in vision-related tracts.

The present study considers a sample of adolescents with dyslexia with and without the intron 2 deletion on the DCDC2 gene. Previous work from our group has suggested the presence of subtle effects of DCDC2 on white matter organization. Here, we test the hypothesis that poor motion perception in DCDC2d+ dyslexia is related to white matter organization in posterior tracks for motion processing by correlating psychophysical performance with white matter integrity.

We did not attempt to perform tractography (Tournier et al. 2012; Pestilli et al. 2014; Takemura et al. 2016; Caiafa and Pestilli 2017) given that no current validated method exists for the correlation between psychophysical performance and bundles parameters assessed by tractography. However, a subtle and local alteration in Fractional Anisotropy (FA, a measure of anisotropy in the diffusivity of water in white matter tracts which reflects fiber density, fiber integrity, and myelination) has been demonstrated in a previous study that correlated behavioral performance with FA in a subpopulation of DCDC2 dyslexics (Marino et al. 2014). The results verified the hypothesis that motion perception correlates with FA abnormality in specific white matter location, in particular several foci along the optic radiation revealed the correlation between FA and motion perception performance. This work reinforces the view that dyslexics DCDC2 carry a specific phenotype with marked visual deficits.

## Materials and methods

### Subjects

Subjects were recruited from an ongoing study which measured prevalence of DCDC2 deletion in a large population of subjects (303 families, corresponding to a total sample of 973 DNA samples). Inclusion criteria were: (1) either accuracy or speed z scores ≤ 2.0 standard deviations on timed text-reading tests (Cornoldi and Colpo 1995, 1998); or (2) either accuracy or speed z scores ≤ 2.0 standard deviations on timed reading of single unrelated words or pronounceable non-word lists (Sartori et al. 1995); and (3) full-scale IQ ≥ 85 (Cattell and Cattell 1981); and (4) absence of neurological or sensorial disorders; and (5) right-handed according to the Briggs and Nebes Inventory (Briggs and Nebes 1975). All subjects who participated in the study had normal or corrected − to − normal acuity, color vision (Ishihara Color Vision Test) and stereo—vision (Frisbee Stereotest). Informed written consent to participate in both the MRI and the psychophysics studies was obtained from 10 probands with and 10 probands without the DCDC2d (hereafter, DCDC2d+ and DCDC2d–, respectively, 5/5 males/females in each group). Age range was 16–21.

Some of these subjects have previously participated to the neuroanatomical study of Marino et al. (2014) and psychophysical study of Cicchini et al. (2015).

### Sample size

A priori power analysis was performed using G-Power Software (Faul et al. 2009). The primary goal of the current research was to leverage on the large variability in motion perception across the whole dyslexic sample to identify anatomical sites of correlation. Considering the low prevalence of DCDC2d+ dyslexics and the difficulty of recruiting them, the current study aimed to demonstrate only the strongest anatomical correlations. Previous work had demonstrated that correlation between behavioral scores and white matter integrity can be as high as 0.5 (Huber et al. 2018). The sign of the correlation also followed a clear prediction (i.e. more disorganization should lead to a perceptual impairment) so we assumed a statistical threshold of 0.05 one tailed. This suggested a minimum of 19 subjects across groups.

### Ethical approval

The protocol was approved by the Scientific Review Board and the Ethical Committee of the “Eugenio Medea” and “San Raffaele” Scientific Institutes.

### Neuropsychological assessment

All subjects were administered the Adult Dyslexia Checklist and several other neuropsychological tests to evaluate reading and reading-related abilities (Vinegrad, 1994; for a detailed description, see Marino et al., (2014)). Briefly, reading, spelling, short-term memory and phonemic awareness tasks were evaluated for all subjects and *z* scores were obtained based on grade/age norms from the general population (Cornoldi and Colpo 1995, 1998; Sartori et al. 1995; Reynolds and Bigler, 1994). *Z* Scores of accuracy and speed in text reading, word reading, non-word reading were averaged to yield a measure of reading proficiency. *Z* scores in syllable displacement, spoonerism, phonemic blending were averaged to obtain a measure of phonemic awareness. Socioeconomic status was based on parental occupation which was scored according to the Hollingshead nine-points scale, whereby a score ranging from 10 to 90 was assigned to each parental job, and the higher of the two scores was used when both parents were employed (Hollingshead 1975). Subjects’ level of education was self-reported as the highest completed grade of high school or year of college at the time of assessment and was analyzed as a continuous variable (hereafter, education).

### Psychophysical measures

The stimuli and procedure employed to measure motion discrimination sensitivity were the same as those of Cicchini et al. (2015), briefly summarized here. Subjects sat in front of a calibrated CRT monitor and were required to discriminate the direction of motion of a brief Gabor patch drifted in the horizontal or vertical direction at 8 Hz. The Gabor was obtained by windowing a sinusoidal carrier by a gaussian envelope which had SD of 2°. The spatial frequency of the sinusoid were 0.5, 1, 2 and 4 c/deg. Presentation was foveal and brief (150 ms) to exclude interference with eye movement. Stimuli were generated by Visage framestore (Cambridge Research System) and displayed on a Sony CRT monitor at 800 × 600 pixels resolution at 120 Hz frame rate, with mean luminance of 35 cd/m^2^. Viewing distance was 57 cm. The physical size of the stimulus was fixed, however, its perceived size (the portion of the stimulus which exceeded visibility threshold) changed with contrast.

**Fig. 1:**
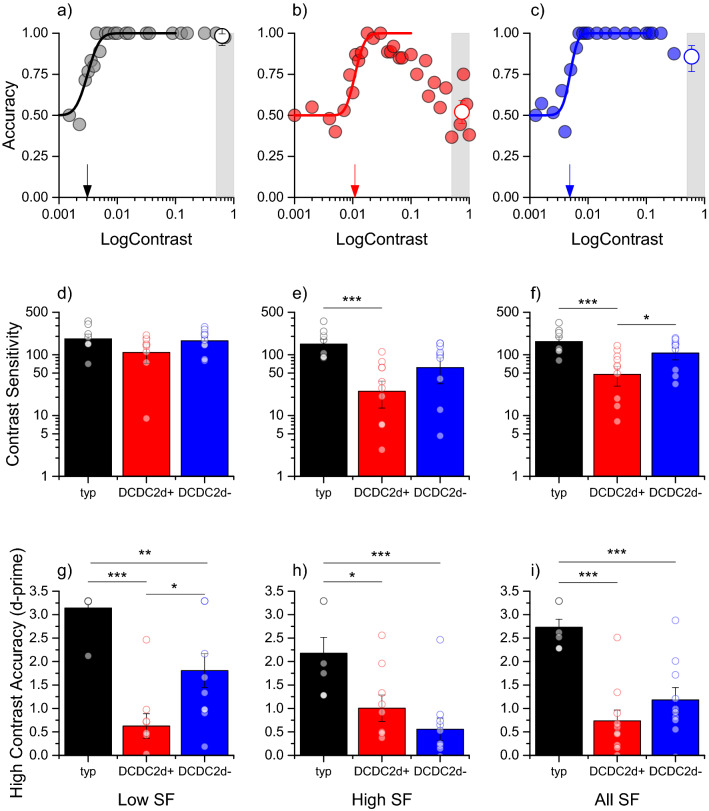
Deficits in motion perception for DCDC2d+ and DCDC2d– dyslexics. **a**–**c** Accuracy for motion direction discrimination as a function of stimulus contrast for a typical observer (**a**), a DCDC2d+ dyslexic (**b**) and a DCDC2d– dyslexic (**c**) for gratings 1 c/deg drifting at 8 Hz. Small arrows indicate the discrimination threshold (i.e. 75% correct responses), the shaded area shows the contrast range (above 50%) used to calculated the high contrast accuracy index (hollow symbol). **d**, **e** Contrast sensitivity for motion in the three groups averaging the low spatial frequencies (0.5 and 1 c/deg (**d**)), the high spatial frequencies (2 and 4 c/deg, (**e**)) or all of the spatial frequencies (**f**). **g**–**i** High-contrast accuracy for the three groups at low (**g**), high (**h**) and all spatial frequencies (**i**). Symbols indicate individual subjects, error bars are S.E.M. Statistically significant differences are flagged (*p < 0.05, **p < 0.01, ***p < 0001)

The subjects reported verbally the perceived direction (one-interval two-alternative forced choice procedure). Stimulus contrast was varied from trial to trial, following an adaptive Quest routine (Watson and Pelli 1983). In cases where the fits were poor, additional trials at specific contrasts were run and added to the dataset. Data were fitted with a cumulative Gaussian psychometric function running from 50 to 100% and the contrast sufficient to yield 75% correct responses is the threshold. Sensitivity is the inverse of threshold. In many subjects the performance at high contrast decreased: in this case the datapoints for the fit were limited to those that yielded a monotonic increase of performance with contrast and skipping those where accuracy fell again (see Fig. 1b for one such examples).

Two psychophysical performances were calculated: (1) contrast sensitivity (i.e. inverse of threshold expressed in logarithmic units) averaged across the two directions of motion (horizontal and vertical); (2) motion discrimination accuracy with a high contrast stimuli (“high contrast accuracy”). This was obtained from the average discrimination accuracy for the stimulus contrast higher than 50% Michelson contrast. To use an appropriate scale for correlations, accuracy values were transformed from percent correct in “d-prime”.

### Magnetic resonance imaging

Magnetic resonance images were collected with a 3 Tesla Philips Achieva scanner (Best, The Netherlands). High-resolution anatomical scans were acquired using a 3D T1-weighed pulse sequence, with the following parameters: TR = 8.06 ms, TE = 4 ms, voxel size = 0.90 × 0.90 × 1 mm, number of slices = 150, matrix size = 245 × 256.

Diffusion images were acquired with EPI DTI pulse sequence and the following acquisition parameters: TR = 9775 ms, TE = 58 ms, sense reduction factor = 2, voxel size = 1.835 × 1.835 × 2.3 mm^3^, *b* = 1000 s/mm^2^, 35 non-collinear directions of the diffusion gradients.

### DTI preprocessing

After correction for Eddy currents and motion, the tensor and FA maps were calculated from DT images using Brainvisa Software (www.brainvisa.info).

White matter structures have been standardized/normalized following a well-validated procedure (Abe et al. 2010). T2 (*b* = 0) images were first spatially normalised using SPM software (https://www.fil.ion.ucl.ac.uk/spm/) to the SPM echo planar imaging template and used for the calculation of normalisation parameters. The corresponding FA maps of each subject were then normalised to this template using the same T2 transformations. As a VB-DTI approach may be sensitive to problems concerning the precise overlap between the same regions in different brains, normalised FA maps of each subject were carefully inspected and successively they were masked to extract only white matter voxels (WM-FA maps). A mean FA template was created averaging the normalized WM-FA maps of all subjects. Finally, the original subject’s FA maps were then normalized again to this new template and then smoothed (6 × 6x6 mm^3^ FWHM). With this improved procedure, we are reassured that we are comparing the same tracts across participants.

Correlations between FA and psychophysical indices were calculated on a voxel-by-voxel basis using SPM software. For the correlations across all subjects of the two groups, we analysed only positive and significant values. For correlations within each group we analysed all correlations that were significantly stronger in the DCDC2d+ group, given that we test the hypothesis that this genetic alteration interferes with early visual pathways. To identify the closest visual area, we mapped the significant cluster in the FSL (www.fmrib.ox.ac.uk/fsl) Jülich and/or Harvard–Oxford cortical atlas.

Finally, to quantify the correlation, we extracted the mean values for each normalised FA map of each subject, inside a sphere of radius 5 mm centered on the maximum of the most significant clusters and correlated it with psychophysical indices calculating the Pearson correlation coefficient.

### Data availability

The data that support the findings of this study are available on request from the corresponding author because of the sensitive nature of the clinical information concerning the participants.

## Results

We measured motion perception in two groups of dyslexics, with (+) and without (–) deletion in DCDC2, matched for age, IQ, and reading disabilities. Figure 1a–c shows sample psychometric curves for a representative typical subject, a DCDC2d+ and a DCDC2d− dyslexic, performing motion discrimination at low frequencies (1 c/deg). Motion perception anomalies occurred in two forms: decrease of contrast sensitivity to motion and reduced accuracy at high contrast. Typical subjects (Fig. 1a) had contrast threshold for direction discrimination around 0.003 (contrast sensitivity of 320), while the DCDC2d+ subject of Fig. 1b had a threshold of 0.01 Michelson contrast (indicated by arrows) which was three-fold higher (contrast sensitivity of 91). Figure 1, bottom row shows motion sensitivity averaging across low (0.5 and 1 c/deg), high (2 and 4 c/deg) and all spatial frequencies tested (Figs. 1d–f). When pooling the data across all spatial frequencies (Fig. 1f), the deficit in motion sensitivity was significantly different from typicals in both dyslexic subject groups with a greater deficit of DCDC2d+ (*t*(16) = 3.29, *p* = 0.005) than DCDC2d– subjects (*t*(16) = 1.60, *p* = 0.013). Importantly, the average sensitivity index was different between the two groups of dyslexic subjects (*t*(18) = 2.2, *p* = 0.04). Confirming previous results (Cicchini et al. 2015), the deficit was not significantly different at low spatial frequencies (DCDC2d+ vs controls: *t*(16) = 1.4, *p* = 0.2, (DCDC2d+ vs DCDC2d– dyslexics (*t*(18) = 1.36, *p* = 0.19).

The deficit of accuracy at high contrasts, which previous literature associated with abnormal intracortical inhibition (Tadin 2015), is particularly interesting. Typically, when stimulus contrast increases, accuracy increases to reach a plateau with near 100% correct responses. However, in typical subjects with very brief exposures (< 30 ms), performance may decrease at high contrast (Derrington and Goddard 1989). Surprisingly, this phenomenon occurred for both dyslexic groups in our paradigm at a much slower presentation time (150 ms: see Fig. 1b, red curve). This resulted in a significant difference in the averaged performance across all spatial frequencies between controls and dyslexics (DCDC2d+ : *t*(15) = 6.7, *p* < 0.0001) with no statistically significant difference between the two dyslexic group accuracies (*t*(18) = 1.2, *p* = 0.22). When averaging the contrast decrement only for the two lowest spatial frequencies, the two groups still performed differently from typicals (*t*(15) = 8, *p* < 0.0001 for DCDC2d+ and *t*(16) = 3.1, *p* = 0.007 DCDC2d–); but importantly, they were also significantly different from each other (*t*(17) = 2.6, *p* = 0.02, Fig. 1f), rendering this index useful to assess potential differences across the two different phenotypes in the correlation with FA.

In conclusion, the two groups of dyslexics subjects had statistically significant different sensitivity when averaging across all spatial frequencies and statistically significant high contrast performance decrement when averaging across the low spatial frequencies. Importantly, these two indices do not exhibit a strong mutual correlation (*r* = 0.42, *p* = 0.26 in the DCDC2d+ group and *r* = 0.32, *p* = 0.36 in the DCDC2d– group) suggesting, in line with the literature, that they are probing different mechanisms. We used these two indices to distinguish the possible alteration in FA across the two groups. Considering that none of them correlated significantly with any summary neuropsychological scores of reading and phonemic abilities in neither group (all *p* > 0.1 uncorrected), any significant correlations between FA and visual motion perception would highlight possible neuronal deficit associated with the motion perception.

Psychophysical and fractional anisotropy indices both have higher values for better performance. Therefore, the psychophysical performance should correlate positively with the FA: better the performance higher the anisotropy. We examined first the population of dyslexics as a whole for a positive correlation, and then we focused on the difference between correlation of dyslexics with and without DCDC2 deletion.

**Fig. 2:**
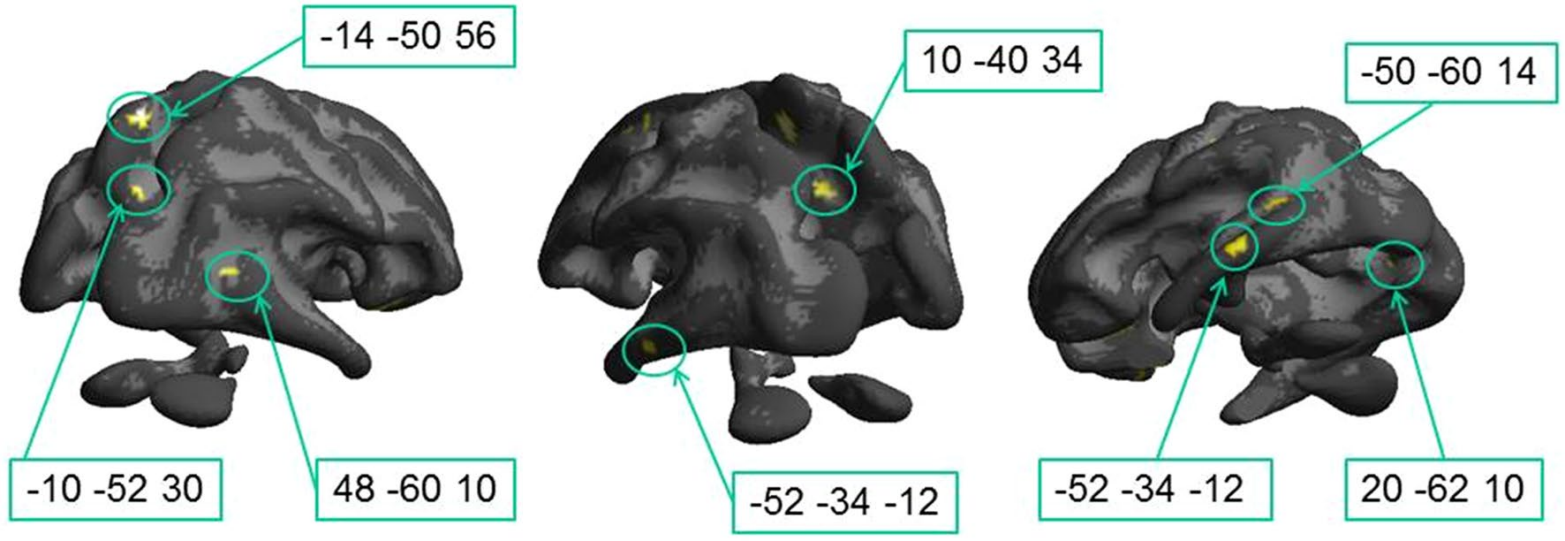
Statistical parametric maps of correlations between fractional anisotropy and motion contrast sensitivity in the whole sample with dyslexia. In this view, only seven statistically significant clusters are visible on the White Matter surface. Other foci are in splenium, optical radiations and inferior temporal gyrus (see Table 1). Threshold: *p* < 0.05 FDR corrected at the voxel level

Figure 2 and Table 1 show voxels in which fractional anisotropy correlated positively with motion contrast sensitivity across the entire dyslexic population. High correlation was present for many white matter tracts nearby the splenium, arcuate fasciculus, superior and inferior longitudinal fasciculi in the temporal region. Importantly, positive and strong correlations existed in key tracks crucial for visual processing of motion, like the two bilateral foci (1C and 1 J) that are located very close to the location of MT (MNI: from (– 48, – 70, − 3) to (– 43,– 60, + 3)) and the two bilateral anterior clusters that are located close to medial part of the Optic Radiation (1B and 1G).

**Table 1:** Posterior Regions (*y* < –30) with significant correlation between fractional anisotropy and motion contrast sensitivity in the whole sample with dyslexia

#	White Matter Brain regions	MNI coordinates							
		Left hemisphere				Right hemisphere			
		*x*	*y*	*Z*	*N* voxels	*x*	*y*	*z*	*N* voxels
1A^§^	Arcuate fasciculus posterior branch	– 14	– 50	56	33				
1B	Optic radiation	– 42	– 46	8	19				
1C^§^	Arcuate fasciculus posterior branch (close to MT)	– 50	– 60	14	17				
1D^§^	Inferior Longitudinal Fasciculus	– 52	– 34	– 12	32				
1E	Inferior Longitudinal Fasciculus near V4	– 38	– 76	– 18	11				
1F^§^	Splenium CC	– 10	– 52	30	13				
1G*§*	optic radiation near peripheral V1					20	– 62	10	14
1H^§^	Splenium CC					10	– 40	34	40
1I	Inferior Longitudinal Fasciculus temporal occipital division					54	– 46	– 26	37
1 J*§*	Inferior Longitudinal Fasciculus near MT					48	– 60	10	18

Only significant regions thresholded at *p* = 0.05, FDR corrected at the voxel level. The regions labelled with §, are illustrated in Fig. 2

**Fig. 3:**
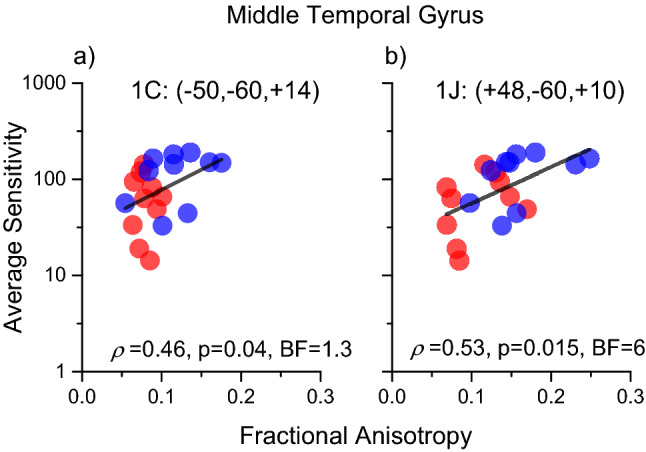
Correlation between fractional anisotropy and motion contrast sensitivity in foci close to MT complex. Sensitivity was averaged across all frequencies. **a** Left hemisphere, Cluster 1C of Table 1; **b** Right hemisphere Cluster 1 J of Table 1. Red refers to Dyslexics carriers of DCDC2 deletion, blue to dyslexics without mutation of DCDC2. Black line shows the correlation across all subjects. Cluster coordinates are in MNI

A previous study (Marino et al. 2014) has shown that the two groups have different FAs in many clusters. Given the significant difference in sensitivity between groups, a significant correlation between FA and motion sensitivity may result from a differential deficit between groups, and not necessarily inside each group. We verified that this indeed was the case for many foci in the visual brain, such as the foci close to MT (Fig. 3). Individual fractional anisotropy values, extracted from the spherical regions (radius 5 mm) centered on the left (Cluster 1C; MNI coordinates: – 50, – 60, + 14, *ρ* = 0.46, *p* = 0.04) and the right (cluster 1 J; MNI coordinates: + 48, – 60, + 10, *ρ* = 0.50, *p* = 0.026) hemispheres close to MT, are plotted against motion sensitivity in Fig. 3: the two groups also have different FAs (1C: *t*(18) = 3.0, *p* = 0.009, 1 J: *t*(18) = 3.2, *p* = 0.008), with DCDC2d+ having lower values and explaining at least partially the high correlation with motion contrast sensitivity.

Other clusters showing significant correlations between FA and sensitivity because of a main effect between groups were also located along the ventral stream at coordinates (1D: – 52,–3 4,– 12), (1E: – 38, – 76, – 18) and (1I: 54,– 46,– 26). In the right hemisphere (1I), these ventral stream foci were located close to Occipital Face Area (OFA) and to Visual Word Form Area (VWFA), while the foci in the left hemisphere (1E) are very close to area V4. All the cortical areas close to these white matter foci are important for visual object recognition and classification, function that may be differentially altered in the two dyslexic populations.

**Fig. 4:**
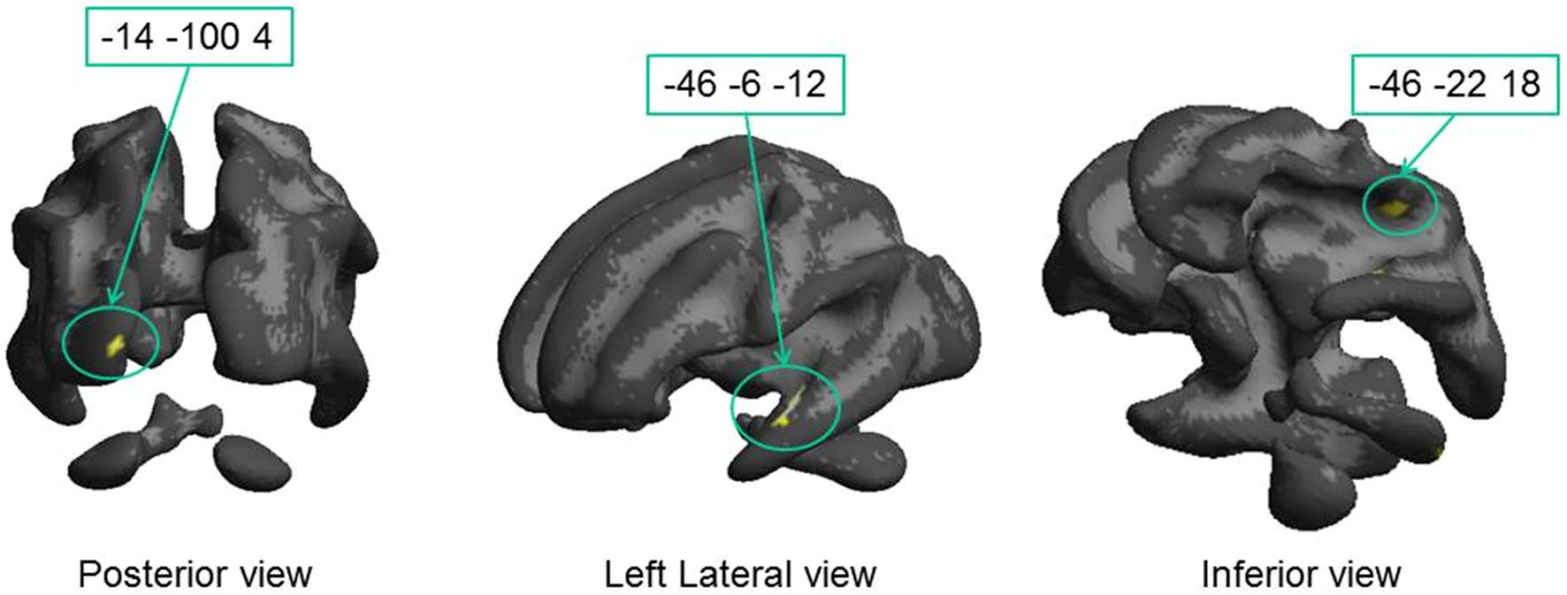
Regions where the correlation between fractional anisotropy and motion contrast sensitivity is significantly higher in the DCDC2d+ group with respect to the DCDC2d– group. Only foci of Table 2 close to visual pathways (2A, 2C and 2D) are displayed on the White Matter surface. Cluster 2B lies deep in white matter and cannot be displayed with this view

To isolate anatomical substrates linked to the genetic trait and to explain the perceptual variance within each group, we mapped regions with significantly higher correlations in the DCDC2d+ group with respect to the DCDC2d– correlations (Fig. 4 and Table 2). This analysis marks both positive and negative correlation in DCDC2d+ , as long as the correlation coefficient in DCDC2d– is lower. This analysis will miss all cases where the two correlations, in sign and magnitude, are very similar.

**Table 2:** Regions with significantly higher correlation between fractional anisotropy and motion contrast sensitivity in the DCDC2d+ group than in the DCDC2d– group

**#**	White Matter Brain regions	MNI coordinates							
		Left hemisphere				Right hemisphere			
		*x*	*y*	*Z*	*N* voxels	*x*	*y*	*z*	*N* voxels
2A^§^	Optic radiations; near V1	– 14	– 100	4	62				
2B	Optic radiations; near V2	– 2	– 98	18	21				
2C^§^	Inferior longitudinal fasciculus, planum polare	– 46	– 6	– 12	99				
2Dlow^§^	Optic radiation	– 40	– 20	0	41				
2Dhigh^§^	Temporal white matter	– 46	– 22	18					
2E	Inferior longitudinal fasciculus	– 62	– 14	– 26	11				
2F	Inferior fronto-occipital fasciculus					34	14	0	14
2G	Inferior fronto-occipital fasciculus					32	8	– 14	14

(*p* < 0.05 FDR, The regions labelled with §, are illustrated in Fig. 4

This analysis highlighted fewer clusters, but interestingly 3 of these were located along the optic radiation. Cluster 2A (62 voxels) was close to primary occipital cortices at coordinates (– 14, – 100, + 4), in correspondence of fiber bundles targeting V1. The other cluster, 2B (21 voxels), was more dorsal and medial (– 2, – 98, + 18) and was located along the dorsal stream in the cuneus inside area BA18 (V2). A third large region with significant different correlation between dyslexic groups was observed in the left hemisphere and comprises two clusters, spanning from coordinates (40, – 20, 0, cluster 2D low) to (– 46 – 22 18, cluster 2D high). The most ventral cluster was located between the elbow of the optic radiations in proximity of the LGN and the Inferior Longitudinal Fasciculus in the frontal region. The most dorsal cluster included large parts of the arcuate fasciculus. Interestingly, all these significant clusters were in the left hemisphere (see Fig. 4), while the closest labelled foci in the right hemisphere were much more anterior and part of the longitudinal frontal-occipital fasciculus.

**Fig. 5:**
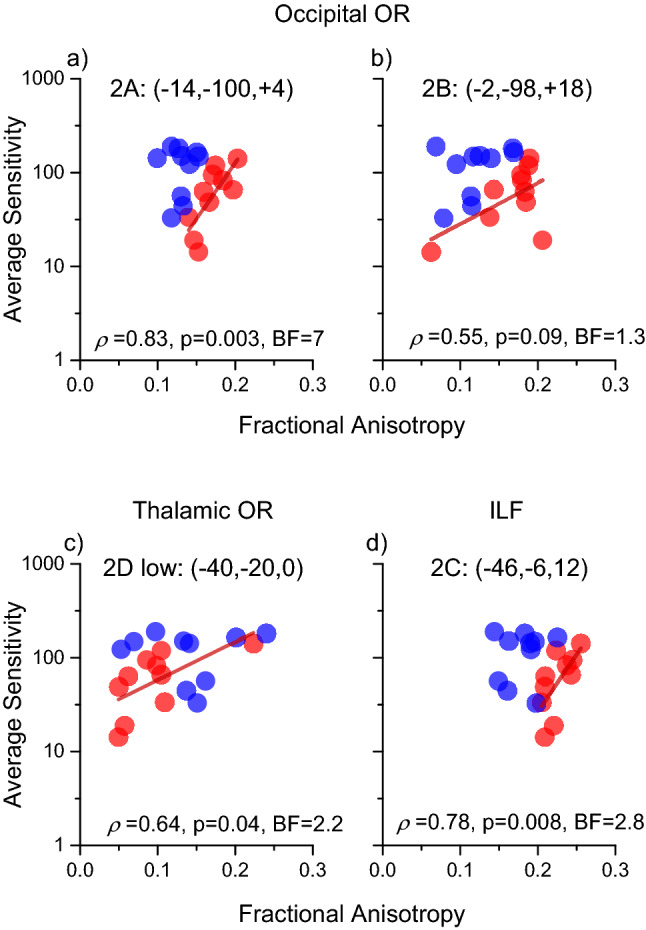
Correlations between fractional anisotropy and motion contrast sensitivity in selected ROIs of Table 2 located within visual cortex (2A, 2B, 2D) and to Planum Polare (2C). Red regression lines show significant associations in the DCDC2d+ group. Correlations in the DCDC2d– group were not significant (2A: *r* = 0.13, *p* = 0.7; 2B: *r* = 0.39, *p* = 0.26; 2D: *r* = –0.02 *p* = 0.95; 2C: *r* = 0.13, *p* = 0.7)

As our analysis is based on a significant difference between the correlation coefficients within the two groups, it is important to show that the correlation between psychophysics and FA in the DCDC2d+ group is positive. To this aim, we plotted individual data contrasting Motion Contrast Sensitivity with fractional anisotropy in representative spherical ROIs (radius 5 mm) centered inside the three clusters of the visual pathways (Fig. 5, the MNI coordinates were referred to the centers of the spheres). The analysis confirms that the correlation was positive and significant for the DCDC2d+ group, while not significant for the DCDC2d– group. It is worthwhile to highlight the 2A and 2B foci, which have strong correlations with the sensitivities and were located in the Optic Radiation (2D; *ρ* = 0.64, *p* = 0.05, BF = 2.2), close to V1 (2A; *ρ* = 0.83, *p* = 0.003, BF = 7) and to V2/V3 (2B; *ρ* = 0.55, *p* = 0.09, BF = 1.3). The correlation was also very strong for a cluster close to the Planum Polare (which processes voice and auditory objects) inside the Inferior longitudinal Fasciculus (2C; *ρ* = 0.78, *p* = 0.008, BF = 2.8).

We observed that not only motion sensitivity is impaired in Dyslexia, but there is also an atypical decrement in performance at high contrast (Fig. 1g–i). The capacity to judge motion direction of brief, high contrast stimuli is a sensitive test of intracortical communication and processing, and has been found to be altered in several pathological conditions such as schizophrenia and autism (Dakin et al. 2005; Snijders et al. 2013; Tadin et al. 2003; Tadin 2015; Yoon et al., 2010).

**Fig. 6:**
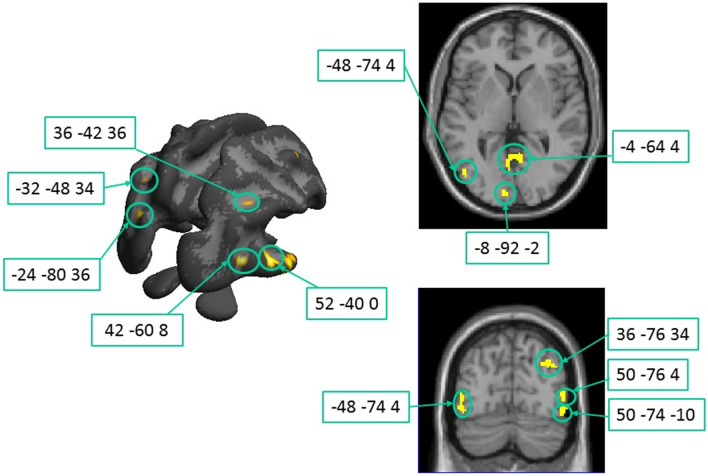
Clusters of correlation between fractional anisotropy and high contrast accuracy across the whole population. Corresponding details are given in Table 3. Threshold: *p* < 0.05 FDR corrected at the voxel level. On the left panel, the clusters are overlapped on the White Matter surface, on the right, the clusters are overlapped on the axial and coronal MR slices for better visualization of the deeper correlation foci

Significant positive correlations between white matter integrity and high contrast accuracy (Fig. 6, Table 3) were revealed in numerous regions were spread across the brain, consistent with a multifaceted etiology of dyslexia. We concentrated the analysis to the foci more posterior than *y* = – 30, which are presumably related to visual processing. The largest cluster resided in the corpus callosum (Table 3, clusters K, but see also clusters E, G and V) suggesting that efficient interhemispheric communication may be a key component for gain regulation at high contrasts across the entire population under study.

**Table 3:** Posterior Regions (*y* < –30) with significant correlation between fractional anisotropy and high contrast accuracy

	White matter brain regions	MNI coordinates							
		Left hemisphere				Right hemisphere			
		*x*	*y*	*Z*	# voxel	*x*	*Y*	*z*	#voxel
3A	Superior longitudinal fasciculus, planum temporale (BA 22)	– 52	– 40	20	136				
3B	Cerebellum	– 26	– 36	– 24	152				
3C*§*	Inferior longitudinal fasciculus near MT	– 48	– 74	4	81				
		– 50	– 74	– 10					
3D*§*	Occipital white matter near V1	– 4	– 64	4	78				
3E*§*	Splenium CC	– 24	– 80	36	98				
3F*§*	Optic radiations near V1	– 8	– 92	2	25				
3G	Splenium CC	– 2	– 60	44	16				
		– 2	60	32					
3H*§*	Arcuate fasciculus close to intraparietal sulcus	– 46	– 60	34	49				
		– 32	– 48	34					
3I	Inferior longitudinal fasciculus close to Wernicke’s area, BA21	– 60	– 34	– 2	69				
3 J	Inferior longitudinal fasciculus near VWFA	– 40	– 54	– 10	10				
3 K	CC body					0	– 34	30	1071
3L*§*	Inf long fasciculus slightly anterior to MT					52	– 40	0	144
3 M	Cerebellum					28	– 40	– 46	141
3 N*§*	Inf long fasciculus near MT					50	– 74	– 10	63
3O*§*	Inf long fasciculus near MT					50	– 76	4	50
3P*§*	Optic radiations occipital pole					42	– 60	8	48
3Q*§*	Arcuate fasciculus					36	– 76	34	70
						36	– 60	40	28
						36	– 42	36	33
3R	Inferior long fasciculus Close to EBA					60	– 62	0	40
3S	Inferior fronto-occipital fasciculus, dorsally to MT					46	– 74	20	59
3 T	Inferior fronto-occipital fasciculus close to FFA					48	– 44	– 24	81
3U	ILF/IFOF					28	– 38	– 6	18
3 V	CC splenium					4	– 56	14	14
3 W	Inf long fasciculus close to MT					62	– 50	– 10	20

Only significant clusters at *p* < 0.05, FDR corrected are reported. The regions labelled with §, are illustrated in Fig. 6

*FFA* fusiform face area, *EBA* extrastriate body area

Many clusters are marked along visual pathways in the occipital, temporal and parietal lobes. One cluster was located close to left medial V1, where optical radiations innervate primary visual areas (Table 3 F; – 8, – 92, + 2). Five clusters were located in the white matter surrounding the MT complex (3C, 3O, 3 N, 3S and 3 W) at locations spanning from (+ 52, – 40, 0), and (+ 50, – 76, 4). A series of clusters were marked along the ventral area and close to the Visual Word Form Area and the OFA, important stations of the reading circuit (3 J and 3 T). Interesting a focus was localized very close to Wernicke area (BA21, 3I).

**Fig. 7:**
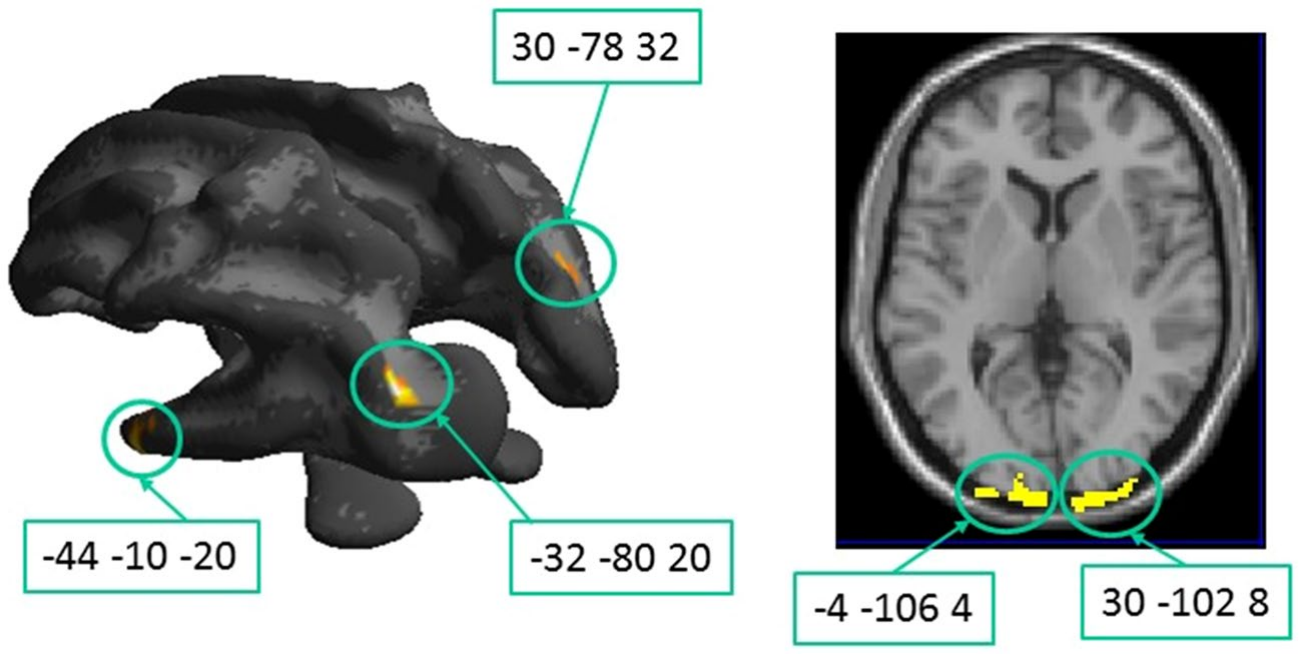
Regions where the correlation between fractional anisotropy and sensitivity at high contrast is significantly higher in the DCDC2d+ vs. DCDC2d− group. On the left panel, the clusters are overlapped on the White Matter surface; on the right panel, they are projected onto an axial slice Corresponding details are given in Table 4

A significant correlation across the whole population may still reflect a generalized impairment of FA and accuracy between the groups, not necessarily explaining the variance across individuals within each group. When searching for higher degrees of correlation within the DCDC2d+ group with respect to DCDC2d–, following the same statistical design of Table 2, only a few regions were marked. Importantly, the marked regions were located in the occipital and parietal white matter bundles related to primary and secondary visual cortical areas and along the arcuate fasciculus (Fig. 7, Table 4). Some of these foci overlap with those marked by the FA *vs* Motion Contrast Sensitivity correlation of Fig. 4, reinforcing the suggestion of their potential involvement in the motion perception deficit.

**Table 4:** Regions with significantly higher correlation between fractional anisotropy and sensitivity at high contrast in the DCDC2d+ vs. DCDC2d– group

	White Matter Brain regions	MNI coordinates							
		Left hemisphere				Right hemisphere			
		*x*	*y*	*Z*	# voxel	*x*	*y*	*z*	#voxel
4A*§*	Inferior longitudinal fasciculus—planum polare	– 44 – 44	– 10 – 8	– 20 – 10	693				
4B*§*	IFOF close to CIP (caudal intraparietal)	– 32	– 80	20	86				
4C*§*	Optic radiations near V1	– 4 – 10	– 106 – 98	4 – 8	246				
4D*§*	Optic radiations near V1					30	– 102	8	228
4E	Arcuate fasciculus					30	– 78	32	80

The regions labelled with §, are illustrated in Fig. 7. (*p* < 0.05, FDR)

**Fig. 8:**
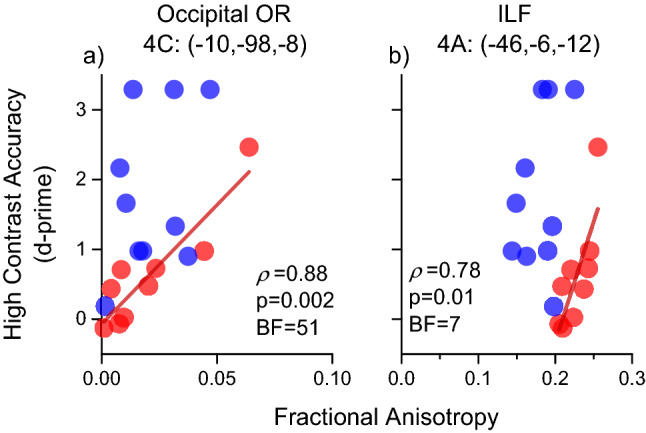
Correlations between fractional anisotropy and high contrast accuracy in selected ROIs of Table 4 belonging to visual pathways (4C) and to the Inferior Longitudinal Fasciculus (4A). Significant positive correlations were found for the DCDC2d+ group, as shown by red regression lines. No significant associations were observed for the DCDC2d– (4C: *r* = 0.38, *p* = 0.27; 4A: *r* = 0.33 *p* = 0.35)

Figure 8 shows two exemplar areas. One is a spherical region extracted from the marked white matter close to primary visual cortex (4C; – 10, – 98, – 8) and not distant from clusters 2A and 2B; all these clusters (4C, 2A and 2B) are part of the optic radiation in the occipital pole. The other cluster (4A) is in overlap with the region of Fig. 5D (cluster 2C; – 44, – 8, – 12), which belongs to the anterior segment of the ILF. Both foci show a strong association between high contrast performance and FA in the DCDC2d+ group, and a non-significant correlation (4C, *r* = 0.38, *p* = 0.27; 4A/2C, *r* = 0.33 *p* = 0.35) with the DCDC2d– group.

## Discussion

Dyslexia is far from being a unitary disorder, and the wide range of deficits associated with it has to be conceived in a multifactorial causal perspective (Pennington 2006). Recently, we showed that a subgroup of adolescents with dyslexia carrying the DCDC2 intron 2 deletion had severe impairments in visual motion perception (Cicchini et al. 2015): the contrast sensitivity impairments for discriminating motion direction were worse by a factor of 10 respect to a control population and by a factor of 3 respect to similarly impaired dyslexic subjects without the DCDC2 mutation.

The present study extends further the results of Cicchini et al. (2015) first by showing that the motion deficit is present also at high contrasts, with the DCDC2d+ population having a drop of motion accuracy for high contrast brief stimuli three times stronger than in the DCDC2d– population; second, by demonstrating for the first time an association of white matter integrity with the impairments of contrast sensitivity and high contrast accuracy of motion direction discrimination in dyslexia. Interestingly, the two psychophysical indexes are not correlated with each other and whilst being statistically significant, also display some overlap between groups suggesting that motion impairments in dyslexics are multifaceted and multifactorial.

The two properties of visual motion perception, which we found to be profoundly impaired in DCDC2d+ dyslexia, are limited by different neuronal mechanisms, and may reveal different neuronal deficits. Motion contrast sensitivity is determined by the spatio-temporal properties of neuronal motion detectors, reflecting sensitivity to contrast from peripheral retinal processes to primary cortical areas. A deficit in contrast sensitivity is considered to reflect impairments in the early stages of visual processing, affecting thalamic inputs and primary visual cortex (Cowey and Gross 1970). For the specific stimuli used here (very brief), we observed a deficit in sensitivity only for the DCDC2d+ population at both high and low frequencies, but not in the DCDC2d– population. It is well know that motion sensitivity is altered in Dyslexia (Lovegrove et al. 1980; Cornelissen 1993; Cornelissen et al. 1995; Lovegrove 1996; Slaghuis and Ryan 1999). However, the present results indicate that the deficits may be associated with specific phenotype, and further suggest that the DCDC2d+ phenotype may have strongly biased the average motion contrast sensitivity impairments found in dyslexia (Martin and Lovegrove 1987; Slaghuis and Ryan 1999).

The second impairment we report is motion direction accuracy at high contrast. This performance is significantly impaired in both populations of dyslexia, being stronger in the one carrying DCDC2 deletion. Although this phenomenon may seem counterintuitive, as more signal leads to worse performance, fine regulation of neuronal gain in visual motion mechanisms is required to achieve accurate perception at high contrasts. Motion detectors receptive fields have center/surround spatial antagonisms and motion discrimination is inhibited when the motion of same direction is presented in the center as well as in the surround of their receptive fields. For this reason, when contrast is high and moving stimuli are large enough to stimulate both the center and the periphery of the RF, direction of motion is perceived less accurately (Tadin et al. 2003; Bhat et al. 2018). The phenomenon that it is commonly observed in typical subjects is exacerbated in neurological conditions, like schizophrenia and autism (Dakin et al. 2005; Snijders et al. 2013), probably reflecting an imbalance between excitatory and inhibitory cortical activities postulated in these pathologies. In the same vein, we might speculate that the deficit, we observed in dyslexia is associated with imbalance between neuronal excitation and inhibitory mechanisms in the receptive fields of motion sensitive neurons. The imbalance would be stronger in DCDC2d+ respect to DCDC2d− population.

The statistically significant differences between motion performance between groups is also associated with statistically significant differences in FA. In a previous paper, we demonstrated that FA in the DCDC2d+ group is lower in many crucial hubs of the visual and reading network (Marino et al. 2014). The perceptual and the FA differences between the two groups explain, at least partially, the highly significant correlation between sensitivity and high contrast versus FA that we observed in many foci. Several ventral and dorsal white matter clusters were located, bilaterally in the inferior longitudinal fasciculus (1D, 3D, 3L, 3R) and in the callosal splenium (1F, 1H, 3E, 3G, 3 K, 3 V), both important bundles connecting many associative visual areas. Many foci (1D, 3 J, 3L, 3R) along the ILF are very close or just superior to the location of the MT complex, which is an important hub in the motion network. Importantly, four foci were located in the optic radiations, suggesting that alterations in dyslexics may be very early in the visual system, with two in proximity of the thalamus. This finding is important, as it shows that poor motion perception in dyslexia is associated with white matter of early projection tracts, connecting the lateral geniculate nucleus to primary visual cortex.

While all these abnormalities are in bundles connecting visual areas, we observed correlations of FA with the motion discrimination in the left posterior branch of arcuate and the superior longitudinal fasciculi, which have been repeatedly associated with language (Catani et al. 2005; Perani et al. 2011) and reading processes (Vandermosten et al. 2012). Again, this result is in agreement with Marino et al., (2014), who found a strong difference in FA at these sites between the two groups. The correlation may result from the stronger FA anomaly in the DCDC2d+ group (which also has stronger motion deficits) and not be linked to the mechanisms that limit the poor performance for motion perception.

Nonetheless, with this caveat in mind, it is interesting to observe that the FA of two bilateral clusters on the inferior longitudinal fasciculus very close to the MT complex correlated both with contrast sensitivity and with high contrast decrement. This result corroborates previous evidence that input–output bundles in MT complex are generally related to reading skills (Ben-shachar et al. 2007), and more generally with the crucial role of MT complex in motion processing. Previous literature has linked the MT area to reading achievement. Functional MRI studies of dyslexics report less BOLD activation in MT compared with controls (Demb et al., 1997; Eden et al., 1996; Heim et al., 2010; but see Olulade et al. 2013). Our findings suggest that part of these impairments are due to damage to white matter fibers targeting MT.

To address more specifically whether the variation in psychophysical performance might be associated with some early visual pathway deficits, we analyzed areas where the DCDC2d+ group showed significantly stronger correlations than the DCDC2d– group. With both psychophysical indices (motion contrast sensitivity and high contrast accuracy), the DCDC2d+ group showed strong associations with white matter in the optic radiations and ventral tracts (inferior longitudinal and inferior fronto-occipital fasciculi), which provide input and output to V1. Importantly, no foci were observed around MT, indicating that the impairment caused by DCDC2 deletion is associated with early visual pathways. Furthermore, we found that only the loss in sensitivity correlated with optic radiation anomalies close to the thalamus, but high-contrast performance did not. This suggests that the DCDC2d+ ’s sensitivity impairment is already determined at thalamic site, while surround inhibition may arise later at cortical level (V1 or V2 given the 4C and 4D foci). This result is consistent with the early report of Galaburda et al., (1985), who observed anatomical deficits of LGN magnocellular cells in post-mortem dyslexic patients. Our results suggest that these subjects might have been carriers of DCDC2 deletion. Recently, the DCDC2d+ alterations have been measured in a mouse knockout animal model (Meng et al. 2005). Animal models of DCDC2 deletion have suggested that DCDC2 gene is important for neuronal migration in utero (Poelmans et al., 2011). Interestingly, Rendall et al., (2017) have demonstrated that in the DCDC2d+ KO mouse LGN neurons are smaller in size, suggesting alteration at the thalamic level. The KO mice are also impaired in motion discrimination, corroborating the influence of the DCDC2 neuronal migration in shaping early visual pathways (Rendall et al. 2017). It would be important to compare FA along the optic radiation tracks between the two groups to reveal possible alteration. There are powerful methods that allows now this quantitative and robust validation (Tournier et al. 2012; Pestilli et al. 2014; Takemura et al. 2016; Caiafa and Pestilli 2017). However, these methods are not yet suitable to study a correlation with psychophysical results.

A limitation of our study is the small sample size. However, the DCDC2d+ subjects are rare and extending to a larger group has proven to be very difficult. Despite this limitation, our results show that DCDC2d+ dyslexic subjects have deficits in very specific structures which are crucial in the relay of visual information and these deficits may have a profound impact during the acquisition of reading skills. Scerri et al. also dismiss the role of DCDC2 in dyslexia also based on their failure to find a difference in coherent motion threshold between the DCDC2 deletion status. However, their conclusion is a simple consequence of the use of not optimal stimuli for assessing motion perception. As described in the introduction, RDK with very long exposure and motion updates are not probing well motion sensitivities and we would encourage the use of more suitable paradigms before dismissing the idea that DCDC2d+ dyslexics constitute a specific subtype of poor readers.

Previous reports in the literature suggest that some of the functional anomalies in dyslexia may be the result of reduced exposure to reading experience (Olulade et al. 2013). Our results are not consistent with this hypothesis. First, the two groups were matched on several variables, including intelligence and reading proficiency. It is thus unlikely that reading habits played a role in one dyslexia group but not the other. Second, several regions of anatomical deficits were located in early visual tracts, for example the optic radiations. These areas mature early in infancy, well before subjects are even exposed to characters. Indeed, our results are consistent with the recent finding that the level of literacy does not play a significant role in psychophysical tests and suggests a biological rather than an environmental cause (Flint and Pammer 2019).

Overall, our findings show that perceptual anomalies of DCDC2d+ dyslexics are accompanied by specific white matter anomalies, many in primary visual pathways, whose integrity is necessary for motion perception. Importantly, many of these fibers mature early in infancy, suggesting that the perceptual deficits should be present also at a younger age. This suggests that the substantial motion deficit observed here, which in some subjects corresponded to a selective motion-blindness, may strongly limit the capability to acquire highly skilled tasks such as reading later on in life. Our results, which point to an early visual deficit, open the way for early diagnosis of this specific dyslexic subtype vulnerabilities, answering the important need to segment dyslexia in sub-types to fully understand the many faced of this complex and heterogeneous disorder.
